# An Experimental Study on the Thermomechanical Coupling Effects of Carbon-Fiber-Reinforced Polyetheretherketone under Dynamic Impact

**DOI:** 10.3390/polym16162295

**Published:** 2024-08-14

**Authors:** Shuyan Nie, Liming Chen, Zhaoxin Yun, Jie Wang, Xin Pan

**Affiliations:** College of Aerospace Engineering, Chongqing University, Chongqing 400030, China; nieshuyan@cqu.edu.cn (S.N.); zhaoxin_yun@163.com (Z.Y.); panxin@cqu.edu.cn (X.P.)

**Keywords:** CF/PEEK, thermomechanical coupling effects, Hopkinson bar impact test, high temperature, microdamage mechanism

## Abstract

Carbon-fiber-reinforced polyetheretherketone (CF/PEEK) composites are widely utilized in aerospace, medical devices, and automotive industries, renowned for their superior mechanical properties and high-temperature resistance. Despite these advantages, the thermomechanical coupling behavior of CF/PEEK under dynamic loading conditions is not well understood. This study aims to explore the thermomechanical coupling effects of CF/PEEK at elevated strain rates, employing Hopkinson bar impact tests and scanning electron microscopy (SEM) for detailed characterization. Our findings indicate that an increase in temperature led to significant reductions in the yield strength, peak stress, and specific energy absorption of CF/PEEK, while fracture strain had no significant effect. For instance, at 200 °C, the yield strength, peak stress, and specific energy absorption decreased by 39%, 37%, and 38%, respectively, compared to their values at 20 °C. Furthermore, as the strain rate increased, the yield strength, peak stress, specific energy absorption, and fracture strain all exhibited strain-hardening effects. However, as the strain rate further increased, above 4000 s^−1^, the enhancing effect of the strain rate on the yield strength and peak stress gradually diminished. The interaction of the temperature and strain rate significantly affected the mechanical performance of CF/PEEK under high-speed impact conditions. While the strain rate generally enhanced these properties, the strain-hardening effect on the yield strength weakened as the temperature increased, and both the temperature and strain rate contributed to the increase in specific energy absorption. Microdamage mechanism analysis revealed that interface debonding and sliding between the fibers and the matrix were more pronounced under static compression than under dynamic compression, thereby diminishing the efficiency of stress transfer. Additionally, higher temperatures caused the PEEK matrix to soften and exhibit increased viscoelastic behavior, which in turn affected the material’s toughness and the mechanisms of stress transfer. These insights hold substantial engineering significance, particularly for the optimization of CF/PEEK composite design and applications in extreme environments.

## 1. Introduction

Short carbon-fiber-reinforced polyetheretherketone (CF/PEEK) composites, known for their superior mechanical properties and high-temperature resistance, have found widespread applications in aerospace, medical devices, and the automotive industry. Numerous studies have investigated the temperature-dependent mechanical properties of CF/PEEK [1,2,3,4], revealing a significant impact of temperature on its performance.

Gaitanelis et al. [5] studied the thermal degradation characteristics and interfacial properties of CF/PEEK, discovering that rapid high-temperature treatment effectively characterized thermal degradation and enhanced the interfacial shear strength (IFSS) by 25%. Almeida et al. [6] employed a thermodynamic viscosity model to determine the degradation-induced solidification limit of CF/PEEK composites, describing viscosity increases due to degradation using a dual Arrhenius law and defining an inherent processing window. Their combined simulations and experimental measurements revealed the effects of changes in processing temperature and pressure on porosity and material properties. Li et al. [2] revealed through continuous dynamic mechanical analysis under multiple temperature scans that the dynamic mechanical properties of CF/PEEK stabilize after several temperature cycles, maintaining a constant glass transition temperature while increasing the peaks of storage modulus and loss factor curves. Despite these valuable insights, deeper research into the behavior and stability of CF/PEEK at high temperatures is necessary, especially for reliability assessments in demanding applications like aerospace and automotive manufacturing, to understand the mechanical property mechanisms under thermal environments.

The strain rate also significantly affects the mechanical behavior of CF/PEEK. Andrew et al. [7] tested the energy absorption and piezoresistive sensing performance of 3D-printed discontinuous CF/PEEK honeycomb composites, finding that under in-plane impact loads, the peak stress and specific energy absorption (SEA) of the CF/PEEK hexagonal lattice increased by 20 and 5 times, respectively, and it displayed clear piezoresistive responses. Tang et al. [8] conducted tensile tests on CF/PEEK at different temperatures and strain rates, showing that tensile strength is highly sensitive to the temperature and strain rate, with failure strain sensitivity significantly decreasing at −30 °C and 100 °C and energy absorption rates reducing at both high and low temperatures. Pan et al. [9] examined the high strain rate compression behavior of woven CF/PEEK thermoplastic composites at 400 to 4000 s^−1^ in glassy and high elastic states, indicating that thermal softening effects surpass strain rate strengthening effects. At low strain rates, out-of-plane impact damage modes transition from the glassy state interface and matrix cracking to high-elastic state “parallel” shearing and fiber crushing, revealing a “fragmentation” shearing mode at high strain rates. Berer et al. [10] studied the dynamic mechanical behavior of PEEK under high-stress stretching, demonstrating excellent mechanical properties and high-temperature resistance under both static and dynamic loads. They recorded strain, load, and surface temperature during fatigue testing and performed quantitative hysteresis loop analysis using calculated chord modulus and dynamic modulus.

Thermoplastic composites, particularly CF/PEEK, exhibit complex thermomechanical coupling behaviors under combined temperature and strain rate conditions. Lei et al. [11] investigated the thermodynamic tensile behavior of PEEK and established a constitutive model, finding that PEEK displays viscoplastic behavior during stretching. Under uniaxial tensile tests at different strain rates and temperatures, the constitutive model can capture necking phenomena related to temperature and the corresponding nominal stress–strain behavior. Sun et al. [12] enhanced CF/PEEK sample quality through preheating and impact compaction processes and analyzed warpage deformation mechanisms using finite element simulations. Current research has started to uncover the dynamic tensile and processing characteristics of CF/PEEK across a range of temperatures and strain rates. However, a comprehensive and systematic investigation into its thermomechanical coupling behavior under high-strain-rate dynamic compression is still required. Therefore, this paper systematically explores the thermomechanical coupling effects of CF/PEEK at high strain rates and various temperatures through comprehensive experiments and microdamage mechanism analyses, providing scientific support and design guidance for the application of CF/PEEK under extreme environmental conditions.

## 2. Experiment

### 2.1. Materials and Specimens

**Figure 1:**
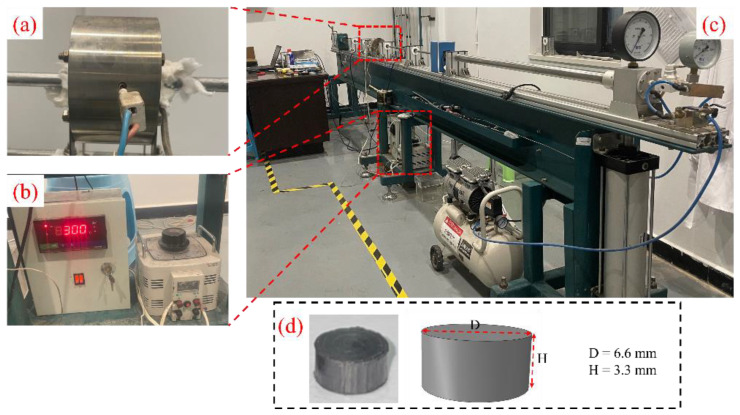
(**a**) High-temperature furnace, (**b**) High-temperature furnace control system, (**c**) High-temperature, high-strain-rate split Hopkinson pressure bar system and (**d**) Specimen sizes.

The short carbon-fiber-reinforced polyetheretherketone (CF/PEEK) composite rods used in this study were produced by uniformly mixing short, chopped carbon fibers with a PEEK matrix and then extruding. The rods were supplied by Dongguan Enbaiyuan Plastic Products Co., Ltd. (Dongguan City, China), with the model PEEK 450CA30. The specific mechanical property parameters are listed in Table 1. The CF/PEEK specimens for testing were machined from these rods, with each specimen measuring 3.3 mm in height and 6.6 mm in diameter, as shown in Figure 1d. The height-to-diameter aspect ratio of the specimen is 0.5 [13,14], adhering to GB/T 34108-2017 [15] and GJB 5982-2007 [16] standards. Experiments were conducted 3 to 5 times for each temperature and pressure setting to ensure the repeatability and reliability of the data.

### 2.2. Experimental Setup

The high-strain-rate compression tests were performed using a high-temperature, high-strain-rate split Hopkinson pressure bar system (SHPB) manufactured by Xi’an Stress Wave Electromechanical Technology Co., Ltd. (Xi’an, China). The split Hopkinson bar system primarily consists of a driving device, striker bar, incident bar, transmission bar, and absorbing bar, as detailed in Figure 1c. Strain gauges attached to the midsections of the incident and transmission bars capture the strains during the impact process via a data acquisition system composed of a computer, velocity measurement device, strain gauges, dynamic strain indicator, and data acquisition card. The incident, transmission, and striker bars all have a diameter of 14 mm, made from 18Ni (C350) martensitic stainless steel, with an elastic modulus $E_{b}$ of 210 GPa, Poisson’s ratio μ of 0.3, density ρ of 7.8 g/cm^3^, and wave speed $C_{b}$ of 5100 m/s. The lengths of the incident and transmission bars are 1200 mm, and the striker bar is 400 mm long. To reduce friction, the contact surfaces between the specimen and incident/transmission bars were evenly coated with petroleum jelly. During the experiment, the specimen was placed inside a high-temperature furnace (as shown in Figure 1a), with a thermocouple wire wrapped around the specimen surface to ensure the set temperature. The high-temperature furnace control system adjusts the heating power based on the temperature measured with the thermocouple to achieve closed-loop control, as illustrated in Figure 1b. The specimen was kept at the set temperature for 5 min. Test temperatures were set to 20 °C, 80 °C, 140 °C, and 200 °C, with air pressure adjusted to 0.05 MPa, 0.075 MPa, and 0.1 MPa to achieve varying stress–strain curves. Before starting the test, the incident bar and transmission bar were closely attached to the two surfaces of the CF/PEEK specimen, and are quickly tested. The SHPB test followed GB/T 34108-2017 standards. After acquiring the incident wave $\varepsilon_{i}(t)$, transmitted wave $\varepsilon_{t}(t)$, and reflected wave $\varepsilon_{r}(t)$ from the SHPB test, the stress–strain curve was plotted using one-dimensional stress wave theory [13,14], which involves the following equations:

(1) $${\dot{\varepsilon }}_{e}\left(t\right)=-\frac{2C_{b}}{l_{s}}\varepsilon_{r}\left(t\right)$$

(2) $$\varepsilon_{e}\left(t\right)=-\frac{2C_{b}}{l_{s}}\int_{0}^{t}\varepsilon_{r}\left(t\right)dt$$

(3) $$\sigma_{e}\left(t\right)=\frac{A_{b}E_{b}}{A_{s}}\varepsilon_{t}\left(t\right)$$

where ${\dot{\varepsilon }}_{e}\left(t\right)$ is the strain rate, $l_{s}$ is the specimen height, $A_{s}$ is the cross-sectional area of the specimen, $A_{b}$ is the cross-sectional area of the incident bar, $\varepsilon_{e}\left(t\right)$ is the engineering strain, $\sigma_{e}\left(t\right)$ is the engineering stress, and *t* is time.

**Figure 2:**
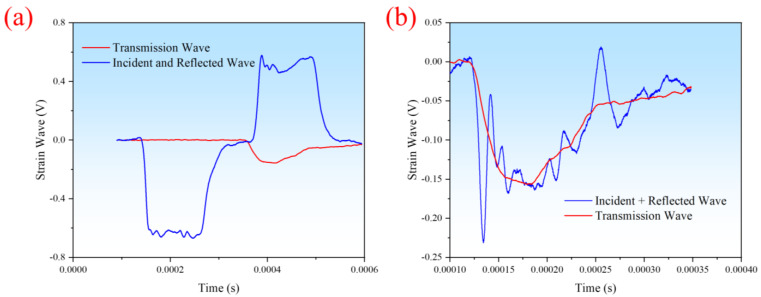
(**a**) Strain waves of SHPB experiments, (**b**) Stress equilibrium verification.

To ensure the reliability of the test data, stress equilibrium verification was performed on randomly selected time–strain waveforms during the experiments. We randomly selected data at a strain rate of 2320 s^−1^ and a temperature of 20 °C for verification, as shown in Figure 2. Figure 2a shows the waveforms of the incident and reflected waves, as well as the transmitted wave. By superimposing the incident and reflected waves and comparing them with the transmitted wave, as depicted in Figure 2b, it can be observed that the waveforms of the superimposed incident and reflected waves coincide well with the transmitted wave. This indicates that the stress reached an equilibrium state, confirming the reliability of the test data.

To investigate the effects of the temperature and strain rate on the material’s mechanical properties, fractured specimens were gold-coated for 2 min to enhance conductivity. Then, they were observed under a scanning electron microscope (SEM) to examine the microscopic fracture morphology. The brand of SEM is Thermo Fisher Scientific (Oregon, OR, USA), and the model is Apreo 2s. The scale for microstructure imaging is 50–500 μm.

## 3. Results and Discussions

### 3.1. Influence of Temperature on Mechanical Properties

**Figure 3:**
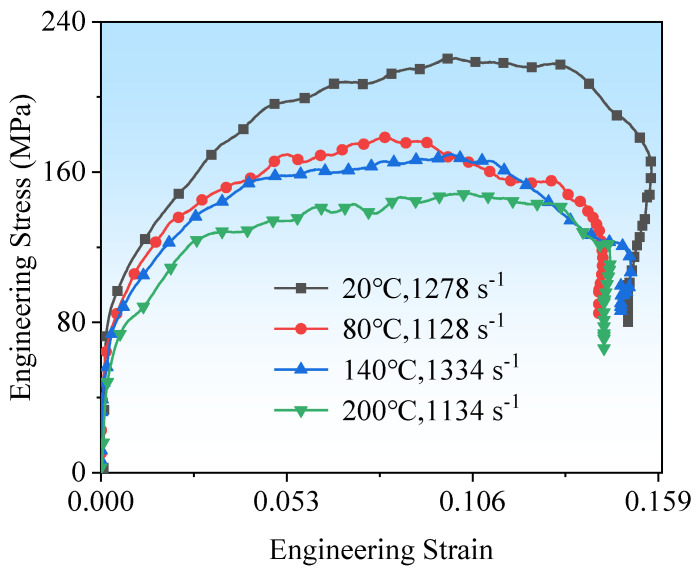
Stress–strain curves at different temperatures at 1200 s^−1^ strain rate.

Temperature is a critical factor reflecting the performance of composite materials, and understanding and controlling its influence on composite materials are essential for optimizing their design and application [17,18]. Figure 3 presents the impact stress–strain curves of CF/PEEK at a Hopkinson bar pressure of 0.05 MPa. The strain rates at 20 °C, 80 °C, 140 °C, and 200 °C are approximately 1200 s^−1^. Similar to ordinary materials, the stress–strain curves of CF/PEEK at different temperatures first exhibit an elastic phase, followed by a plastic phase where stress increases with strain, showing evident strain hardening. With an increase in temperature, the slope of the elastic phase decreases, the elastic modulus reduces, and the peak force declines. After the elastic phase, the material enters a damage phase where stress begins to decrease until fracture occurs.

**Figure 4:**
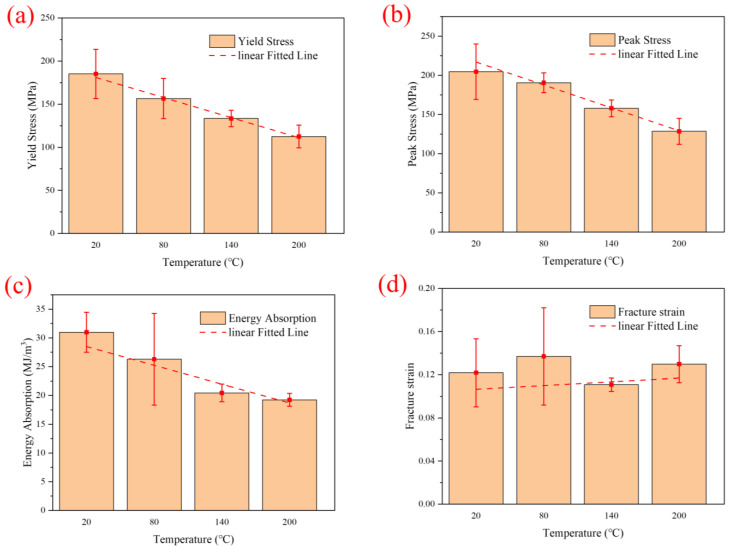
(**a**) Yield strength, (**b**) peak stress, (**c**) Specific energy absorption, and (**d**) fracture strain at 1200 s^−1^ strain rate.

The yield strength, peak stress, specific energy absorption, and fracture strain are important indicators for evaluating the impact mechanical properties of composite materials [19]. To further explore the temperature effects of CF/PEEK at high strain rates, the yield strength, peak stress, specific energy absorption, and fracture strain at a strain rate of 1200 s^−1^ were fitted, as shown in Figure 4. The yield strength was obtained from the linear turning point of the stress–strain curve, specific energy absorption from the area enclosed by the stress–strain curve and the *x*-axis, peak stress from the maximum stress value, and fracture strain from the plastic strain value at material fracture. Figure 4a–c show that the yield strength, peak stress, and specific energy absorption decrease with increasing temperature, generally following a linear trend. Figure 4d indicates that the plastic fracture strain follows a linear trend with temperature, yet its magnitude remains almost constant. It is evident that the mechanical properties of CF/PEEK decrease with an increase in temperature, but material ductility increases, which is associated with matrix softening and reduced load-bearing capacity at high temperatures.

The *R*^2^ values for the linear fitting of the yield strength, peak stress, specific energy absorption, and fracture strain are 0.9935, 0.9834, 0.8387, and 0.0653, respectively. The R-squared (*R*^2^) is the coefficient of determination, which serves as a metric for assessing the goodness of fit of a regression model. It represents the proportion of the variance in the dependent variable that is explained by the model. The *R*^2^ value ranges between 0 and 1, with a value closer to 1 indicating a better fit of the model. The formula for calculating *R*^2^ is as follows:

$$R^{2}=1-\frac{SS_{res}}{SS_{tot}}$$

where *SS_res_* refers to the sum of squared residuals, and *SS_tot_* refers to the total sum of squares.

Additionally, the yield strength, peak stress, and specific energy absorption have the best linearity. Table 2 shows the experimental results of the yield strength, peak stress, specific energy absorption, and fracture strain of CF/PEEK at high temperatures, as well as their comparative values with normal temperatures. At 200 °C compared to 20 °C, the yield strength, peak stress, and specific energy absorption decrease by 39%, 37%, and 38%, respectively, while fracture strain increases by 7%. The yield strength, peak stress, and the degree of energy absorption reduction are almost identical. At 140 °C compared to 20 °C, the yield strength, peak stress, and specific energy absorption decrease by 28%, 23%, and 34%, respectively, while fracture strain decreases by 9%. Thus, at higher temperatures, the yield strength, peak stress, and specific energy absorption all decrease significantly with an increase in temperature, and they are all highly sensitive to the temperature [20,21].

### 3.2. Influence of Strain Rates on Mechanical Properties

**Figure 5:**
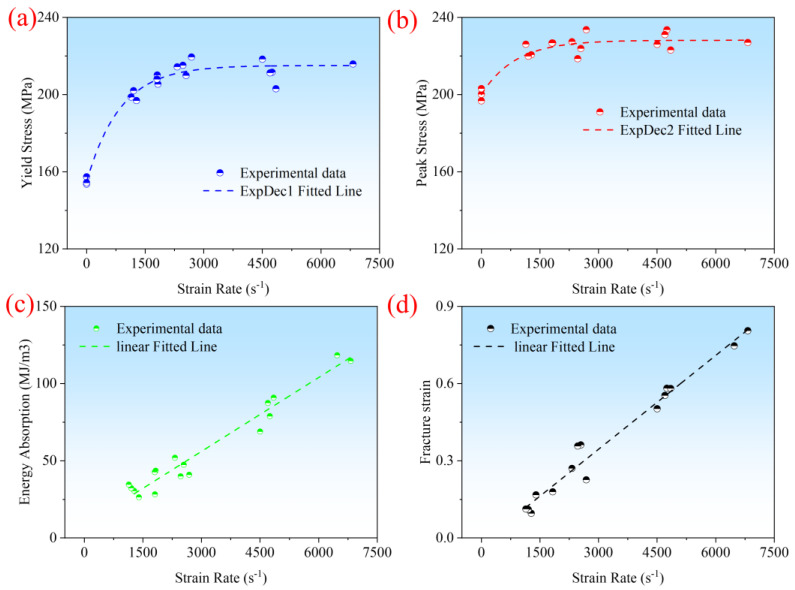
(**a**) Yield strength, (**b**) Peak stress, (**c**) Specific energy absorption, and (**d**) fracture strain at different strain rates under room temperature conditions [19].

**Figure 6:**
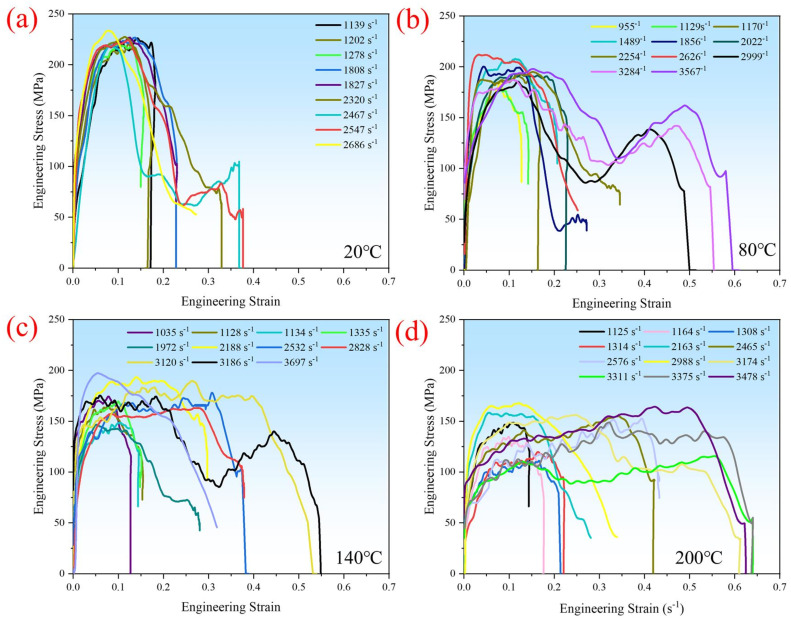
Engineering stress–strain curves at (**a**) 20 °C, (**b**) 80 °C, (**c**) 140 °C and (**d**) 200 °C.

The strain rate, a parameter describing the deformation speed of materials under stress, significantly impacts their mechanical behavior and properties. The strain rate strengthening effect is a crucial characteristic of material impact dynamics, while high-temperature resistance is a vital indicator for the service environment of materials. CF/PEEK is renowned for its excellent impact resistance, making the study of its impact performance crucial for expanding its application fields [22,23]. Figure 6a shows the impact stress–strain curves of CF/PEEK at room temperature under Hopkinson bar pressures of 0.05 MP, 0.075 MP, and 0.1 MP, corresponding to strain rates of 1278 s^−1^, 1827 s^−1^, and 2686 s^−1^, respectively. At room temperature, at strain rates of 1278 s^−1^ and 1827 s^−1^, the stress in the plastic phase of the CF/PEEK stress–strain curve strengthens with increasing strain until a fracture occurs. However, at a strain rate of 2686 s^−1^, the enhancement effect weakens as strain increases. Figure 5 displays the changes in the yield strength, peak stress, specific energy absorption, and fracture strain of CF/PEEK at room temperature with the strain rate. To more effectively elucidate the underlying patterns, we introduced relevant experimental data [19] for comparative analysis. The materials used in the experiments reported in the literature [19] share identical processes, models, and batches with those in this paper. The distinction between the tests in this paper and those reported in the literature lies in the specimen dimensions. The ratio of height to diameter for the specimens in this paper is 0.5, while it is 1.0 in the literature. However, both dimensions fall within the range of 0.5 to 1.0 for the ratio of height to diameter, as required by the relevant literature [13,14] and the GB/T 34108-2017 standards. It can be seen that increasing the strain rate generally enhances yield strength, peak stress, specific energy absorption, and fracture strain. The relationship between the yield strength, peak stress, and strain rate can be described by a two-stage exponential decay function. With an increase in the strain rate, the strain rate strengthening effect diminishes, becoming insignificant when the strain rate exceeds 4000 s^−1^ for yield strength and peak stress. The specific energy absorption and plastic fracture strain exhibit a linear trend with an increase in the strain rate.

It is evident that CF/PEEK exhibits significant enhancement with an increase in the strain rate. At room temperature, the strain rate enhances the yield strength, peak stress, specific energy absorption, and fracture strain. Notably, when the strain rate exceeds 4000 s^−1^, the enhancement effects on the yield strength and peak stress become insignificant. The compression impact curves under high strain rates exhibit strain-hardening effects that reduce as the strain rate increases.

### 3.3. Coupling Effects of Temperature and Strain Rate

Under the simultaneous influence of the temperature and strain rate, the impact dynamics characteristics of materials undergo significant changes, often exhibiting coupling effects [24,25]. Based on the analysis of the strain rate and temperature on the impact dynamics of CF/PEEK in the previous two sections, this section discusses the coupling effects under the joint action of the temperature and strain rate. The stress–strain curves obtained from the compressive impact tests of CF/PEEK vary under different thermal environments. Figure 6 shows the stress–strain curves at different strain rates and temperatures. At each strain rate, the initial stage of the CF/PEEK stress–strain curve is an elastic phase, followed by yielding, then passing through a plastic phase before a fracture occurs. Around a strain rate of 1200 s^−1^, the plastic phase exhibits a strain-hardening effect; that is, as the strain increases, the stress continuously rises until material damage occurs after reaching maximum stress, as seen in the curves for 1278 s^−1^ at 20 °C, 1129 s^−1^ and 1489 s^−1^ at 80 °C, 1335 s^−1^ at 140 °C, and 1164 s^−1^ at 200 °C. When the strain rate is greater than 2000 s^−1^, the plastic phase displays a plateau effect, such as the curves for 2547 s^−1^ at 20 °C, 2188 s^−1^ and 3284 s^−1^, and 2828 s^−1^ at 140 °C, and 2163 s^−1^ and 3174 at 200 °C. Therefore, it can be seen that at 20 °C, 80 °C, and 140 °C, as the strain rate increases, the plastic phase of the CF/PEEK stress–strain curve gradually transitions from strain hardening to strain weakening. This is because a large amount of heat is generated during high-speed impact, causing localized temperature rise in the material and reducing its yield strength, thus leading to strain weakening [5,26]. At 200 °C, with an increase in the strain rate, the degree of transition from strain strengthening to strain weakening slows down. This is because the glass transition temperature (Tg) of CF/PEEK is 143 °C, and 200 °C is greater than 143 °C. Above Tg, the mobility of the polymer matrix’s molecular chains increases, leading to the increased viscoelasticity of the material, thereby reducing the response to strain strengthening. This increased segmental mobility aids in stress relaxation and plastic deformation [27,28,29]. The combined action of strain rate and temperature causes the plastic phase of the material’s stress–strain curve to be influenced not only by the strain rate but also by the temperature. The specific manifestation of the coupling effect of the temperature and strain rate is as follows: the strain rate diminishes the strain-hardening effect during the plastic phase of the material’s stress-strain curve. However, as the temperature rises, this diminishing effect is progressively mitigated.

**Figure 7:**
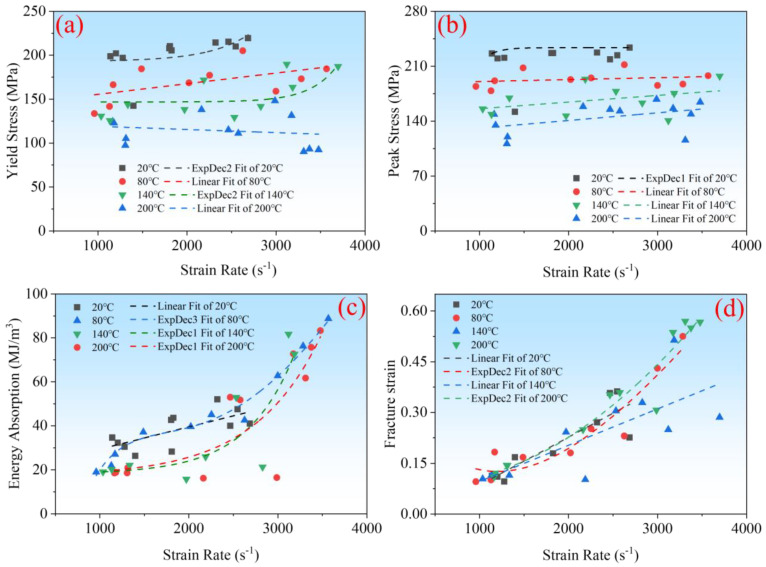
The relationship between the (**a**) Yield strength, (**b**) Peak stress, (**c**) Specific energy absorption, (**d**) fracture plastic strain and temperature under different strain rates.

**Figure 8:**
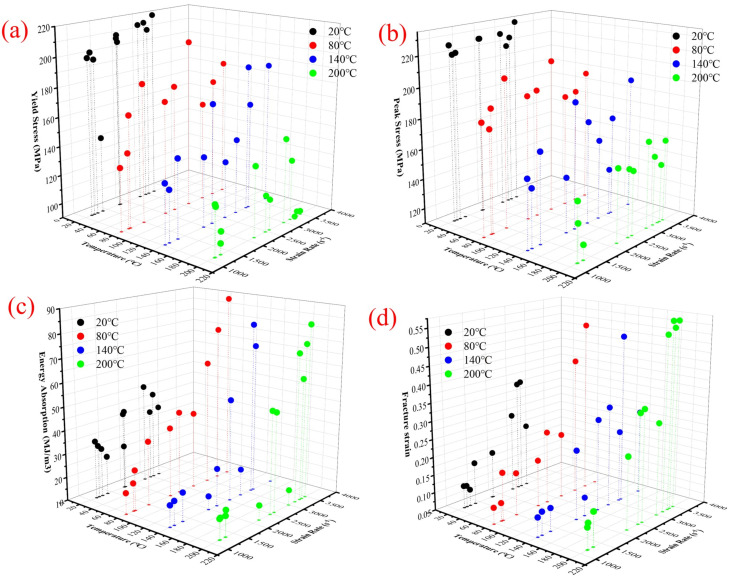
(**a**) Yield strength, (**b**) Peak stress, (**c**) Specific energy absorption, and (**d**) Fracture plasticity at different temperature and strain rate conditions.

In this study, we investigated how the yield strength, peak stress, specific energy absorption, and fracture strain of CF/PEEK vary with different strain rates. Figure 7 displays the characteristics of these metrics for CF/PEEK at various strain rates, while Figure 8 shows how these metrics change with both the strain rate and temperature. From Figure 7a and Figure 8a, it is evident that as the strain rate increases, the yield strength of the material gradually rises, exhibiting a clear strain rate strengthening effect. At 20 °C and 140 °C, when the strain rate increases from 1000 s^−1^ to 4000 s^−1^, the yield strength can be described by a two-stage exponential decay function. As shown in Figure 5a, with an increase in the strain rate, its strengthening effect diminishes, and when the strain rate exceeds 4000 s^−1^, the enhancement of the yield strength due to the strain rate is no longer significant at 20 °C. When the strain rate increases from 1000 s^−1^ to 4000 s^−1^, at temperatures of 80 °C and 200 °C, the yield strength can be described by a linear elastic function with respect to the strain rate. However, at 80 °C, the yield strength exhibits a strain-hardening effect. In contrast, at 200 °C, the strain-hardening effect of the yield strength is not pronounced. The relationship between the yield strength and strain rate at 20 °C and 140 °C can be described by a two-stage exponential decay function, whereas at 80 °C and 200 °C, a linear description is used. This indicates that the temperature alters the trend of interaction between the yield strength and strain rate. The slopes of the fitting curves at 80 °C and 200 °C indicate that at 80 °C, the slope is positive, while at 200 °C, the slope is near zero and negative. This suggests that the temperature has a mitigating effect on the strain rate strengthening effect. Therefore, the specific coupling effect of the yield strength with the strain rate and temperature manifests as follows: the strain rate has a strengthening effect on the yield strength, but as the temperature increases, this strengthening effect gradually decreases, and the trend of the effect changes.

Figure 7b and Figure 8b illustrate the changes in peak stress with varying strain rates and temperatures. It is observed that as the strain rate increases, the material’s peak stress gradually rises, demonstrating a clear strain rate strengthening effect. Similar to the yield strength, at 20 °C, when the strain rate increases from 1000 s^−1^ to 4000 s^−1^, the peak stress can also be described by a two-stage exponential decay function. As shown in Figure 5a, above a strain rate of 4000 s^−1^, the peak stress remains almost constant. However, at 80 °C, 140 °C, and 200 °C, when the strain rate increases from 1000 s^−1^ to 4000 s^−1^, there is a noticeable rise in peak stress, which can be approximated by a linear fit. This indicates that temperature similarly alters the trend of interaction between the peak stress and strain rate. The slopes of the fitting curves from 80 °C to 200 °C do not show a significant trend of increase or decrease. It can be observed that as the temperature rises, the trend of the effects of strain rate and peak stress is altered, with the strain-hardening effect persisting and showing no significant change with the increase in temperature.

Figure 7c and Figure 8c provide statistical data on the specific energy absorption as a function of the strain rate and temperature. When the strain rate increases from 1000 s^−1^ to 4000 s^−1^, the specific energy absorption of the material exhibits a strain-hardening effect. At 20 °C, unlike the yield strength and peak stress, the specific energy absorption can be described by a well-fitting linear elastic model. At 80 °C, the specific energy absorption can be fitted to a three-stage exponential decay function. At 140 °C and 200 °C, with the increase in the strain rate, the specific energy absorption can be fitted to a single exponential decay function. This indicates that the temperature also changes the interaction trend between the specific energy absorption and strain rate. Observations of the fitted curves show that at 140 °C and 200 °C, the strain-hardening effect on specific energy absorption is more pronounced than at 80 °C. This suggests that within the range of 20 °C to 200 °C, there is a strain-hardening effect on specific energy absorption, and the effect becomes more pronounced with the increase in temperature.

Figure 7d and Figure 8d display the experimental results for fracture strain as a function of the strain rate. With an increase in the strain rate, the material’s fracture strain demonstrates a strain rate strengthening effect at all temperature points. When the strain rate increases from 1000 s^−1^ to 4000 s^−1^, at 20 °C and 140 °C, the fracture strain can be fitted with a dual exponential decay function with respect to the strain rate. At 80 °C and 200 °C, the fracture strain can be fitted with a linear function. The temperature does not have a significant effect on the trend of the strain rate’s influence on the fracture strain.

In summary, the combined effects of the strain rate and temperature lead to the plastic phase of the material’s stress–strain curve being influenced not only by the strain rate but also by the temperature. As the temperature rises within the range of 20 °C to 200 °C, the trend of the effects of the strain rate on the peak stress, yield strength, specific energy absorption, and fracture strain is altered. The strain rate has a strengthening effect on the yield strength, but this effect gradually diminishes as the temperature increases. The strain rate also has a strengthening effect on the specific energy absorption, and this effect becomes more pronounced with the increase in temperature. At different temperatures, the strain-hardening effect on the peak stress and fracture strain persists, and the degree of this hardening effect does not change significantly with the increase in temperature.

### 3.4. Microdamage Mechanisms

**Figure 9:**
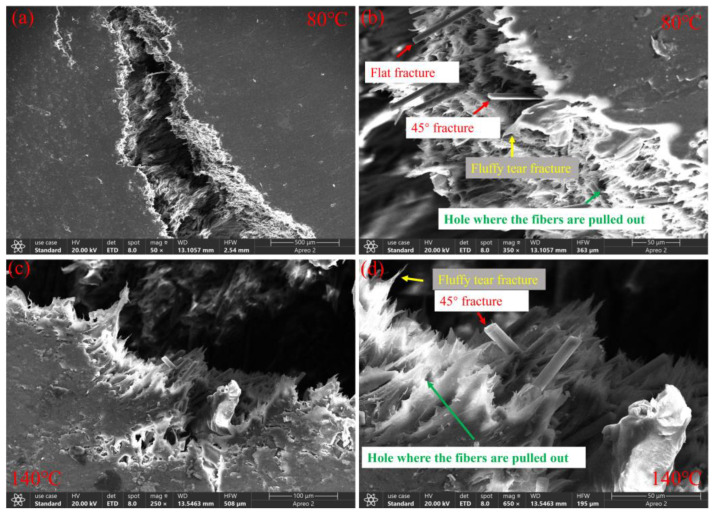
Three-dimensional morphology images under static compression at (**a**,**b**) 80 °C and (**c**,**d**) 140 °C.

**Figure 10:**
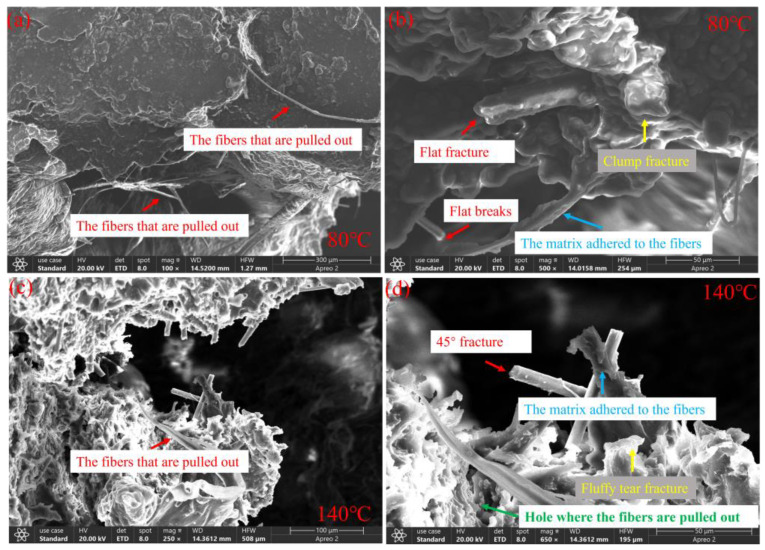
Three-dimensional morphology images under compressive impact at strain rates of 2500 s^−1^ with (**a**,**b**) 80 °C and (**c**,**d**) 140 °C.

The mechanical behavior of CF/PEEK is significantly influenced by the temperature and strain rate, necessitating a detailed analysis of these factors. To analyze the effects of different temperatures and strain rates on its yield strength, peak stress, specific energy absorption, and fracture strain, we compared the microscopic morphology images obtained from SEM. Figure 9 shows the three-dimensional morphology images under static compression conditions at 80 °C and 140 °C. Figure 10 displays the three-dimensional morphology images under dynamic compressive impact conditions at 80 °C and 140 °C. With the temperature increasing from 80 °C to 140 °C, significant changes occurred in the internal microstructure of the material. Comparing Figure 9a,b with Figure 9c,d, it can be seen that under static loading, at both 80 °C and 140 °C, the PEEK matrix forms a fluffy tear fracture, as indicated by the yellow arrows in the figures, with the fracture at 140 °C being longer. This indicates that due to the influence of temperature, the PEEK matrix softens, leading to better toughness at 140 °C, similar to a viscoelastic effect [23,30]. This viscoelastic behavior allows the material to deform to a certain extent when subjected to impact or load, thereby enhancing its toughness [31]. At room temperature, PEEK is in a glassy state, and its yield stress increases with the strain rate. However, near Tg, in the high-elastic state, the yield stress and elastic modulus of the material decrease [32], resulting in a decrease in strength at high temperatures [33].

Comparing Figure 10a,b with Figure 10c,d, it can be seen that at 80 °C and 140 °C, dynamic loading at a strain rate of 2500 s^−1^ causes the PEEK matrix to adhere to the surface of the carbon fibers, as indicated by the blue arrows in the figures. Compared to dynamic loading, static loading results in more interfacial debonding and sliding phenomena between the fiber and matrix, which reduces stress transfer efficiency and makes peak stress more sensitive to the strain rate. At 80 °C, dynamic loading at a strain rate of 2500 s^−1^ results in a large amount of PEEK matrix adhering to the fiber surface in a lump-like manner, more than at 140 °C, and the surface is smoother and rounder, as shown in Figure 10a,b. This indicates that under high-speed impact, the adhesion degree of the PEEK matrix to the fibers at 140 °C is greater than that at 20 °C. It can be seen that near Tg, the viscoelastic effect of PEEK is more significant.

Furthermore, the SEM images in Figure 9 and Figure 10 show that during material failure, holes are formed due to fiber pull-out, as indicated by the green arrows in the figures. Also, under static loading at 80 °C, most fibers exhibit flat breaks, with a few breaking at 45°, as shown by the red arrows in Figure 9b. At 140 °C, most fibers break at 45°, with a few exhibiting flat breaks, as shown by the red arrows in Figure 9d. This indicates better toughness of the carbon fibers at 140 °C. Under dynamic loading at a strain rate of 2500 s^−1^, at 80 °C, a large area of fibers is completely pulled out without breaking, as shown by the red arrows in Figure 10a,b, with the rest of the broken fibers showing flat breaks. At 140 °C, a few fibers are completely pulled out, as shown by the red arrows in Figure 10c, with the rest of the fibers being sheared off, mostly breaking at 45°. This indicates that under static compression, the material is subjected to a slowly applied load, leading to internal stress concentration and subsequent fracture. Under high-speed impact conditions, an increase in the strain rate leads to an improvement in the material’s fracture toughness [34], with the toughness enhancement of the PEEK matrix being less than that of the carbon fibers, resulting in a significant overall fiber pull-out effect. At 140 °C, the overall fiber pull-out effect is less than that at 80 °C. This shows that as the temperature increases, the stress transfer mechanism of CF/PEEK changes. At lower temperatures, the matrix material is harder, easily generating higher shear stress at the interface between the fiber and the matrix, leading to easy fiber pull-out. At high temperatures, the modulus of the matrix material decreases, allowing for more uniform stress transfer to the carbon fibers and thus reducing the phenomenon of fiber pull-out.

## 4. Conclusions

This study investigated the high-speed compressive impact behavior of CF/PEEK at elevated temperatures through Hopkinson pressure bar impact tests, and by analyzing the SEM fracture morphology, the following conclusions were drawn:

(1) As the temperature increases, the yield strength, peak stress, and specific energy absorption of CF/PEEK decrease, while the fracture strain does not show significant changes.
(2) With an increase in the strain rate, the yield strength, peak stress, specific energy absorption, and fracture strain all exhibit strain-hardening effects. However, as the strain rate further increases, above 4000 s^−1^, the enhancing effect of strain rate on the yield strength and peak stress becomes less pronounced.
(3) The temperature and strain rate have a coupling effect on the high-speed impact mechanical properties of CF/PEEK. The strain rate has an enhancing effect on the yield strength, peak stress, specific energy absorption, and fracture strain. As the temperature rises, the strain-hardening effect on the yield strength weakens, while the strain-hardening effect on the specific energy absorption is enhanced.
(4) The analysis of microscopic damage mechanisms reveals that compared to dynamic compression, static compression results in more interfacial debonding and sliding phenomena between the fiber and the matrix, leading to reduced stress transfer efficiency and thus making the peak stress highly sensitive to the strain rate. The increase in temperature causes the PEEK matrix to soften, enhancing viscoelastic behavior, which in turn affects the material’s toughness and stress transfer mechanism.

## Figures and Tables

**Table 1:** The material parameters of CF/PEEK.

Materials	Young’s Modulus (GPa)	Tensile Strength at 20 °C (MPa)	Density (g/cm^3^)	Fiber Diameter (μm)	Fiber Length (μm)	Weight Percentage of Short Fibers	Glass Transition (°C)	Melting Point (°C)
CF/PEEK	20.00	265.00	1.40	4–8	40–150	30%	143	343

**Table 2:** Experimental results of the yield strength, peak stress, specific absorption energy, and fracture strain at 1200 s^−1^.

Temperature (°C)	20	80	140	200
Yield strength (MPa)	185	157	133	112
Compared to 20 °C, the yield strength increased by (%)	0	−15	−28	−39
Yield strain (%)	6	3	4	6
Compared to 20 °C, the yield strain increased by (%)	0	−49	−40	−7
Specific energy absorption (MJ/m^3^)	31	26	20	19
Compared to 20 °C, the specific energy absorption increased by (%)	0	−15	−34	−38
Peak stress (MPa)	205	191	158	129
Compared to 20 °C, the peak stress increased by (%)	0	−7	−23	−37
Fracture strain (%)	12	14	11	13
Compared to 20 °C, the fracture strain increased by (%)	0	+12	−9	+7

## Data Availability

The original contributions presented in the study are included in the article, further inquiries can be directed to the corresponding authors.
